# What do we know about some of the most conspicuous scorpion species of the genus *Tityus*? A historical approach

**DOI:** 10.1186/s40409-015-0016-9

**Published:** 2015-06-10

**Authors:** Wilson R. Lourenço

**Affiliations:** Département Systématique et Evolution, Muséum national d’Histoire naturelle, UMR7205, CP 053, 57 rue Cuvier, Paris, 75005 France

**Keywords:** Scorpion, *Tityus*, Noxious species, Historical aspects

## Abstract

In the present study, comments are proposed on historical aspects of the most conspicuous scorpion species of the genus *Tityus* found in Brazil. Both *Tityus bahiensis* (Perty) and *Tityus serrulatus* Lutz & Mello are better known for their infamous reputation of noxious species. However, the original discovery and description of both species are associated with interesting historical episodes. A short comment is also provided on *Tityus costatus* (Karsch), the species possibly involved in the first record of a scorpion incident in Brazil.

## Introduction

Even if non-scientists may consider scorpions fascinating animals, their interest is in general limited to the bad reputation of these animals as “killers of men”. Nonetheless, a limited number of species – probably no more than 50 – are actually implicated in serious or lethal incidents. Indeed, this relatively small number of species possesses venoms with potent toxins, capable of killing humans. However, it is true that these infamous scorpion species are responsible for an important number of human deaths every year, which is only surpassed by those caused by snakes and bees [1, 2].

Most deadly species belong to the family Buthidae C. L. Koch. Nevertheless, species belonging to at least two other families, Scorpionidae Latreille and Hemiscorpiidae Pocock, also include a few other scorpions that may pose threat to humans.

The origin of mammal-specific toxins appears as an important issue in scorpion evolution. Old World lineages of Buthidae with very potent neurotoxic venom, such as the genera *Androctonus* Ehrenberg and *Leiurus* Ehrenberg, share separate mammal- and insect-specialized neurotoxins, which are specific for Na + channels [2]. Inversely, New World genera, including *Centruroides* Marx and *Tityus* C. L. Koch, have potent toxins acting on both mammals and insects. As an example, it was showed that one of the most potent anti-mammal toxin from *Tityus serrulatus* venom, called TsVII or Tsϒ, is also highly effective against insects [3]. It is quite possible that the separate mammal-specific Na + toxins could have evolved during aridification of the Palaearctic in the Tertiary period, when one of the most important selective factors was rapid spread of small burrowing mammals (mostly rodents) in arid landscapes. Such newcomers to the scorpion environment, rodents would be direct competitors for space (burrows) and important nocturnal predators, as many of them are today [4]. Such pressure explains emergence of specific mammal-targeting toxins, used for defense not for hunting [5].

The family Buthidae comprises around 100 different genera – some currently extinct – and more than 1000 species, corresponding to 50 % of all presently known scorpions. Among these genera, the Neotropical *Tityus* is by far the most conspicuous one, with near to 220 described species. Only two other genera of buthids show almost equally important numbers, *Ananteris* Thorell and *Centruroides* Marx, each with approximately 80 described species.

The aim of this work is to provide some information about the genus *Tityus*, in general and about some of its most remarkable species in particular. The subject is addressed in the form of a historical approach. The targets are readers who possibly use these organisms in their own research, but are not aware of their biological diversity. For this reason, both the text and illustrations are prepared to ensure clarity, accuracy and unambiguous communication, hopping that in this way the matter may be accessible to a broad audience.

## The genus *Tityus* C. L. Koch, 1836

The genus *Tityus* was first created by C. L. Koch in 1836 [6] having as type species, by monotypy, *Scorpio bahiensis* Perty, 1833 [= *Tityus bahiensis* (Perty, 1833)]. This means that when Perty [7] described the species *Scorpio bahiensis*, he placed it in the old genus *Scorpio* Linnaeus, 1758. In fact, *Scorpio* is the original genus within the order Scorpiones, and subsequently to Linnaeus’ work [8], many other species were accommodated in it. However, with the advance of scorpion taxonomy, only *Scorpio maurus* Linnaeus, 1758, the original species described by Linnaeus [8] was maintained in this genus.

The type locality indicated by Perty [7] for *Scorpio bahiensis* [= *Tityus bahiensis* (Perty, 1833)] was “*Habitat prope Bahiam*”, presumably in Brazil, since the material he studied was originally collected during the early 19^th^ century expedition performed in this country by J. B. von Spix and C. F. Ph. von Martius. This locality remains, however, extremely imprecise. I will return to this point in the next section dedicated to *Tityus bahiensis*.

Since the creation of the genus *Tityus*, a large number of new described species was placed in it. Consequently, early revisions concerning the status of different species proved to be a need. A pioneer work is the one produced by Karl Kraepelin [9] in his *Das Tierreich*. This contribution was important because it was carried out in the context of a world revision of all scorpions known up to that date. Although this contribution was extremely valid at that point, covering all the 34 known species of *Tityus*, it could not be final. In fact, for several of these species Kraepelin [9] could only count on very limited samples that invalidated any analysis of the stability or variability of the taxonomic characters used by him.

Subsequently to Kraepelin’s contribution [9], new species and subspecies were described in a fast pace, leading to the necessity of new revisions. Besides, having into account the increasing complexity of the genus *Tityus*, several authors indicated the need to divide it into several groups of species, in some cases, artificially. Kraepelin [10] had already stated that the genus *Tityus* was the most complex among the American genera of scorpions owing to its abundance of species and the difficulties to find useful characters allowing the definition of natural groups.

He attempted himself to create three groups of species using the pigmentation pattern as a guide. Large size species with blackish-brown pigmentation (group *cambridgei*), species of moderate size with dark longitudinal stripes (group *bolivianus*) and species of small size with variegated pigmentation (group *columbianus*). Unfortunately, he observed a degree of variation in pigmentation patterns and reached the conclusion that this character was not entirely satisfactory. However, at the same time, he stated that he could not find other characters!

Following the work of Kraepelin, other authors attempted to revise the genus *Tityus*. Mello-Campos [11] proposed an identification key to the species, without, however, any division of the genus in natural groups of species. More solid attempts to divide the genus in groups of species finally come in the publications by Mello-Leitão [12, 13] and particularly in his monographic work on all South American scorpions published in 1945. In that study, Mello-Leitão [14] proposed a synthesis of all his previous results and the genus *Tityus* was divided into 15 *Formenkreise* (what literally means ‘circles of morphs’ or natural groups). Subsequently, from 1945 until 1975, numerous papers on the genus *Tityus* have been published, but no change to this classification was proposed.

The matter concerning groups of species – or sometimes complexes – within *Tityus* began to be discussed again by the end of the 1970s, in particular by Lourenço [15–21]. Nevertheless, any attempt to proceed with a global revision of the genus proved to be extremely complicated, mainly because of the constant increase in the number of new species as observed by Lourenço [22–27]. A major attention was then dedicated to the interpretation of the observed patterns of distribution and differentiation of several groups of species [28–32]. At the same time, the number of new described species has experienced a constant increase [33–40]. Consequently, the number of described species of *Tityus* currently reaches almost 220, which is far from being final.

By the early 2000s, the issue on the division of the genus *Tityus* in groups of species was discussed again and to a certain degree Lourenço [34] restored the proposition of Kraepelin [10], by retaining three major groups of species, based mainly on pigmentation patterns, but also on some morphological characteristics. Each of these groups was named after the oldest species belonging to the group, which achieved the following results: species group *Tityus clathratus*, species group *Tityus bahiensis* and species group *Tityus asthenes*. Two other new insights were equally proposed in this period. The description of the new genus *Caribetityus* Lourenço for the *Tityus* species distributed in the Greater Antilles, an issue not always accepted by other authors, and the proposition of a new species group for some particular species of *Tityus* found in the Amazon canopy [41–43].

Considering all these novelties, as well the polemics among several authors, I finally decided to propose a division of the genus *Tityus* in several subgenera. The objective was to bring further stabilization to the classification of the group [44]. The genus *Tityus* was than divided into five subgenera. Three of them were based on already available genus-group names. Two new subgenera were created: *Archaeotityus* Lourenço to accommodate the species previously associated with *Tityus clathratus* group, and *Brazilotityus* Lourenço that accommodates some Amazonian canopy species. The composition of the genus *Tityus* in five subgenera remains as follows: *Archaeotityus* Lourenço comprising the *Tityus clathratus* group of species; *Tityus* C. L. Koch containing the *Tityus bahiensis* group of species; *Atreus* Gervais composed of the *Tityus asthenes* group of species; *Caribetityus* Lourenço encompassing several *Tityus* species of the Greater Antilles; and *Brazilotityus* Lourenço comprising the *Tityus adisi* group of species. My decisions concerning the composition of the genus *Tityus* are still a matter of polemics among certain authors and are rejected by others. Nevertheless, on account of the complexity of this group of scorpions, this decision appeared as fundamental. The *Tityus* species considered in the following sections belong all to the subgenus *Tityus*.

## *Tityus bahiensis* Perty, 1833

**Fig. 1:**
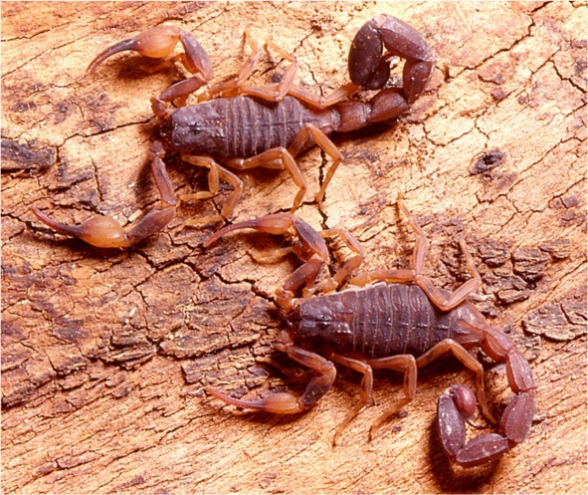
Male and female of *Tityus bahiensis* from the state of São Paulo, Brazil

*Tityus bahiensis* (Fig. 1) was described by Perty in 1833 [7] as *Scorpio bahiensis* [= *Tityus bahiensis* (Perty, 1833)]. According to the summarized biography proposed in Papavero [45], Joseph Anton Maximilian Perty was born in Ohrnlau (Duchy of Anspach) in 1804, and died in 1884. He studied medicine and natural sciences in Landshut and Munich. Afterwards, he joined the Faculty of Sciences of Munich and was in charge of the organization of part of the zoological collections of the city’s Academy of Sciences. Among the valuable collections of the academy, there was the one brought home by Drs. Johann Baptist von Spix and Karl Friedrich Philip von Martius from their long journeys in Brazil, financed by King Maximilian Joseph I. The results of Perty’s studies of this collection were published in a large *folio*, from 1830 to 1833 (including the description of *Scorpio bahiensis* in 1833). In 1833, Perty was named professor of the Academy of Berne in Switzerland and transferred to the University in the next year. He became also the Dean of the University from 1837 to 1856.

As already mentioned, the type locality indicated by Perty [7] for *Scorpio bahiensis* [= *Tityus bahiensis* (Perty, 1833)] was “*Habitat prope Bahiam*”, presumably in Brazil since the material he studied was originally collected during the early 19^th^ century expedition performed in the country by J. B. von Spix and C. F. Ph. von Martius. This locality remains, however, extremely imprecise and led to subsequent misinterpretation.

**Fig. 2:**
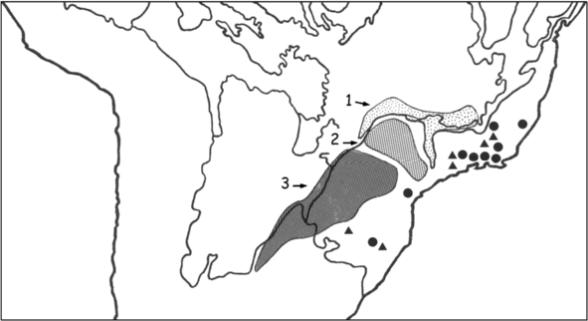
Map of the South and Southeast regions of Brazil showing the distribution of *Tityus bahiensis*. Numbers 1 to 3 indicate northern and southern morphs. *Tityus costatus*: *trifasciata* form (●) and *maculata* form (▲)

**Fig. 3:**
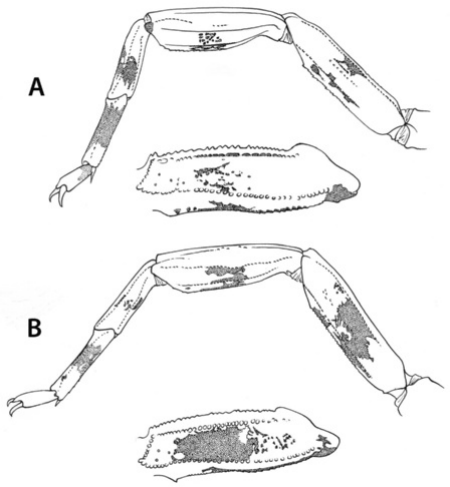
Leg IV and pedipalp femur of *Tityus bahiensis*, showing the distinct pigmentation patterns observed in the morphs distributed in the (**a**) northern and (**b**) southern range of distribution

Another point of interest is the fact that *Scorpio bahiensis* (= *Tityus bahiensis*) was the very first Brazilian scorpion to be described. It was later transferred to the genus *Tityus* by Koch [6]. The type locality indicated by Perty [7] as “*Bahiam*” does not really mean that the type locality is from the state of Bahia, as incorrectly suggested by several authors and in particular by Mello-Leitão [14]. In fact, the original range of distribution of this species does not include Bahia (Fig. 2). The specimen collected by Spix and Martius, used in the original description of *T. bahiensis*, was deposited in the Zoologische Staatssammlung, Munich, Germany, but was destroyed during World War II. Consequently, any verification of the characteristics of the original specimen is therefore impossible. Lourenço [17] observed that individuals from the northern range of distribution presented differences in the pattern of their pigmentation when compared with those from the southern part of the range (Fig. 3). At first, I believed that different species were involved. However, further investigation across the whole gradient indicated that *T. bahiensis* is a polymorphic species.

**Fig. 4:**
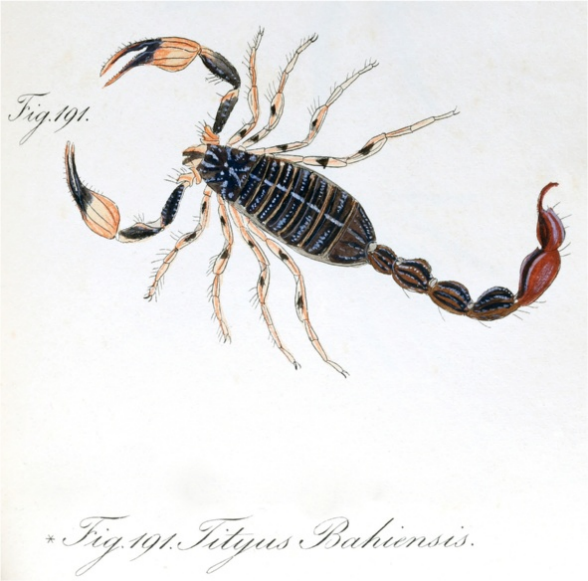
Color illustration of *Tityus bahiensis* presented in the original publication by C. L. Koch [6]

Even if the original specimen used in the description is destroyed, it was very precisely illustrated in colors, both by Perty [7] and C. L. Koch [6] (Fig. 4). These original pictures suggest that the original specimen corresponds rather to the forms found in the southern range of distribution of the species.

The description of the journey of Johann Baptist von Spix and Karl Friedrich Philip von Martius in Brazil may bring some clarification about sites from where the original specimen of *T. bahiensis* was possibly collected. Parts of this text, as reported in Papavero [45], are presented below.

The Bavarian naturalists Karl Friedrich Philip von Martius (born 17 April 1794 in Erlangen, northern Bavaria; died in Munich, 13 December 1868) and Johann Baptist von Spix (born 9 February 1781 in Höchstadt; died 14 March 1826 in Munich), after some weeks in Rio, where they made the acquaintance of Krusenstern and Langsdorff, decided to initiate their trip through Brazil. Through Baron von Neveu they obtained permission to enter the Brazilian Provinces. They collected in the city of Rio de Janeiro (Laranjeiras, Corcovado, Aqueduto, Fonte da carioca – where the English Consul, Mr. Chamberlain, an amateur entomologist, had a coffee plantation –, Tijuca, Botafogo, Jardim Botânico). Invited by Langsdorff, they spend some days at the Farm Mandioca, and continued for a distance on the road to Minas Gerais, to a farm not too far from the Rio Paraíba. Upon their return to Rio, informed by Count von Wrbna that they could not stay in Brazil for more than two years, they decided to go immediately on their expedition. After acquiring mules for the transportation of the equipment, they went through Itaguai (13 December 1817), Areais (already in the Province of São Paulo), Lorena, Guaratingueta, Aparecida, Pindamonhangaba, Taubaté, Jacareí, Mogi das Cruzes, arriving in the City of São Paulo on 31 December 1817. In São Paulo, they met Count von Wrbna, Thomas Ender, Prince von Thurn und Taxis, and Count von Palfy, who had come with the Archduchess’ train. Leaving São Paulo on 9 January 1818, they proceed by way of Cotia, São Roque, Sorocaba (whence they sent collections of natural history to São Paulo and Rio), Ipanema (now Varnhagen) Porto Feliz, then returned to Sorocaba, and proceeded to Itu, Jundiaí, and Atibaia, all in the Province of São Paulo. Entering Minas Gerais, Spix and Martius journeyed through Camanducaia, crossed the Rio Sapucaí, continued along São Gonçalo (do Sapucaí), Campanha (14 February, 1818), crossed the Rio do Peixe and the Rio Grande, passing by São João del Rei, and arrived at the then capital of the Province of Minas Gerais, Vila Rica (Ouro Preto). From Vila Rica they went to visit a village of the Coroado Indians, and afterwards continued their trip via Mariana (21 April 1818), whence they visited the Serra do Caraça, and returned on the 28^th^ to Vila Rica. Through the Governor, Manuel de Portugal e Castro, they dispatched the collections gathered in the itinerary between Sorocaba and Vila Rica. On 1 May 1818, they left the capital of the Province, and went on to Sabará, Caeté (at the time also called Vila Nova da rainha), Vila do Principe (Sêrro), Tejuco (Diamantina), Fanado or Bom Sucesso (presently Minas Novas), Montes Claros, and Contendas (Brasília de Minas), where they remained for some time, for that region was very rich in zoological specimens, and left on 12 August 1818. Following a N-NE direction, they crossed the Rio São Francisco and the Rio Carinhanha, entering the present State of Bahia (at the time a part of the Province of Pernambuco) […] [45].

The long expedition of Spix and Martius reached the present states of Bahia, Piauí and Maranhão, after they got permission to visit the Province of Grão-Pará. Their research was carried out in many regions of the present states of Pará and Amazonas and their journey in Brazil lasted until 13 June 1820, when both embarked on the ship Nova Amazona back to Europe. Their collections, sent to Munich, via Hamburg, consisted of 3381 species of animals of which 80 belonged to Arachnida (including the specimen of *T. bahiensis*).

Only the first part of their expedition, covering the states of São Paulo and Minas Gerais, may be related to the collection of the original *T. bahiensis*. However, the colored illustrations supplied by both Perty [7] and C. L. Koch [6] (Fig. 4) correspond quite well to the forms found in the southern range of distribution of the species. Therefore, it can be suggested that the original specimen was collected in one of the visited sites in the state of São Paulo.

Morphologically, *Tityus bahiensis* is well known. The following general diagnosis can be proposed: scorpions of medium to large size, ranging from 55 to 68 mm in total length, and dark brownish general coloration with reddish-yellow legs and reddish-brown pedipalps. The presence of dark spots on the pedipalps and legs is more marked in specimens from the southern range of the population, i.e. Argentina, Paraguay, and São Paulo state in Brazil, whereas those from certain parts of the states of Goiás and Minas Gerais have only small spots (Fig. 3). Metasomal segments I to III are usually reddish-brown, whereas IV and V are darker, blackish-brown. Dentate margins of pedipalp-chela fingers are composed of 17 oblique rows of granules, the subaculear tooth is strong and spinoid, and their pectines have 18 to 23 teeth. Their geographical distribution includes the Brazilian states of Minas Gerais, Goiás, São Paulo, parts of Mato Grosso do Sul and Paraná; as well as Argentina and Paraguay.

## *Tityus serrulatus* Lutz & Mello, 1922

**Fig. 5:**
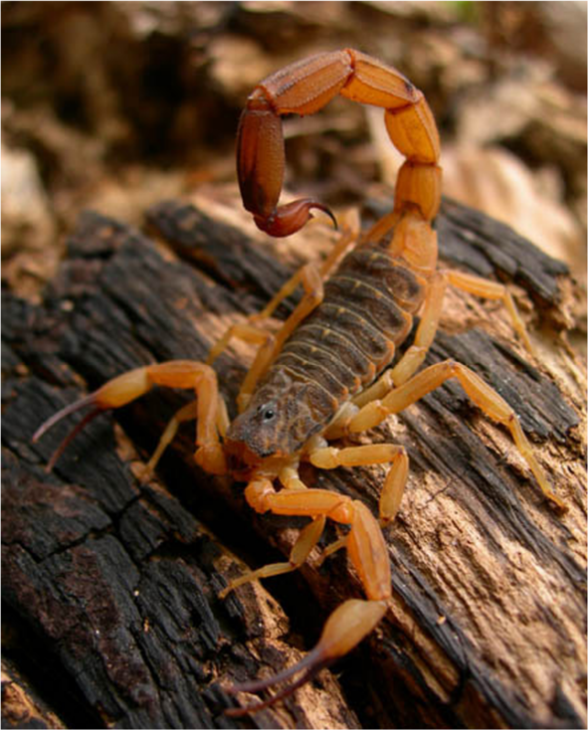
Female of *Tityus serrulatus* from the state of Bahia (photo courtesy of T. J. Porto)

In a short note devoid of any illustrations, Lutz and Mello [46] described five new species of buthid scorpions, among them *Tityus serrulatus* (Fig. 5), a medium-sized yellow scorpion. To this new species, they indicated Belo Horizonte as type locality and mentioned that the type specimen was deposited in the collections of the Instituto Ezequiel Dias. Attempts to find the type specimen of *T. serrulatus* proved to be vain, suggesting that the specimen was damaged or lost. This situation conducted Lourenço and Eickstedt [47] to designate a neotype (a replacement type), based on material equally collected in Belo Horizonte (as recommended by the International Code of Zoological Nomenclature). This neotype was deposited in the collections of the Instituto Butantan in São Paulo, but was probably destroyed during the recent fire incident that took place in the collection’s building of this institution [48, 49].

**Fig. 6:**
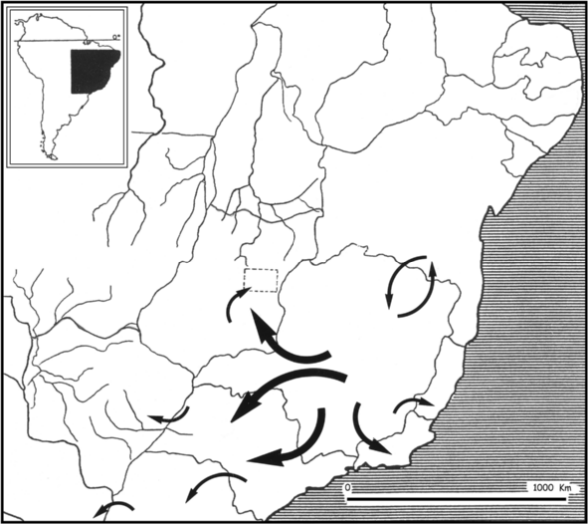
Map of the Southeast and Central regions of Brazil. Arrows illustrate the expansion of *T. serrulatus* over a period of several decades

Today it is well known that the geographic distribution of *T. serrulatus* (the Brazilian yellow scorpion) has expanded considerably over the Southeast, South and Central regions of Brazil (Fig. 6). Everyone also agrees that this species poses major health problems due to its rapid expansion in urban areas, rapid proliferation and great toxicity. However, the possible history of the evolution of *T. serrulatus* in Brazil, and even in parts of South America, is, to say the least, singular.

Scorpionism was known in Brazil in the end of the 19^th^ century and has been documented since then. The first comprehensive study of the phenomenon was the one by Maurano [50], who concentrated his observations on *Tityus bahiensis*, the second most noxious scorpion species in Brazil.

Nevertheless, before the contribution by Maurano [50], not much had appeared in the literature about scorpion problems in Brazil. This particularity seems curious when compared to the enormous health problems caused by scorpions currently. In addition, in other countries such as Mexico, where scorpionism is also a severe health problem, this phenomenon has been cited regularly in the literature since the 16^th^ century [51]. More recent studies include those by Clavigero [52] and Duges [53]. Moreover, the scientific and medical aspects of this issue have been the subject of detailed studies since the beginning of the century [54–56].

The fact that the work carried out by Maurano [50] in 1915 dealt only with *Tityus bahiensis* and that antivenom serum against this species has been produced since 1915 is probably related to the fact that *Tityus serrulatus*, which was only described in 1922, was much less common in the early 20^th^ century than today. However, one major question arises: how can such a common species as *T. serrulatus –* well known since the 1920s (according to Magalhães [57], his laboratory in Minas Gerais received 600.000 specimens between 1922 and 1952) – had not been observed previously and was described rather later than other common species?

Even if the main particularity of *T. serrulatus* has always been its noxious character and medical importance, one other point remained enigmatic during several years: the absence of males from all known populations. The question was finally answered by Matthiesen [58], who first demonstrated that this species could reproduce by parthenogenesis. This phenomenon was later confirmed for a few other species of scorpions, but remains rare [59]. *Tityus serrulatus* was usually considered an obligate parthenogenetic species, but some isolated bisexual populations have finally been detected [60, 61]. However, bisexual populations appear to be extremely marginal within the present geographic distribution of the species, which suggests their possible elimination from most, if not all, the unpredictable environments occupied by *T. serrulatus* [62, 63].

It was previously suggested by Lourenço and Cloudsley-Thompson [60] that *T. serrulatus* could be closely related to *Tityus stigmurus* (Thorell), a bisexual species with a range of distribution further to northeastern states of Brazil. However, the final discovery of sexual populations of *T. serrulatus* invalidated this hypothesis. On the other hand, a previously described and more southern distribution of *T. stigmurus* – which resembles the present geographic range of *T. serrulatus* – was accepted by some authors [11, 64]. By checking the personal notes of J. Vellard and some of the material that he collected in the late 1920s, it can be proposed that *T. stigmurus* was a possible common species in the state of Minas Gerais and south of Goiás, at least until the 19^th^ century. It is possible, therefore, that both species have been misidentified during several years, since *Tityus stigmurus* was described by Thorell [65].

The precise period during which parthenogenenesis appeared *de novo* within the population of *T. serrulatus* is difficult to establish. However, the presence of this species in Minas Gerais before the beginning of the 18^th^ century was probably extremely inconspicuous. At the beginning of the 18^th^ century, an important development was engendered by the Portuguese (especially in their search for gold) with the foundation of towns such as Curral d’El Rei and Vila Rica de Ouro Preto [66]. Previously, metaclimax environments suffered human impact, turning them into disclimax. This favored the previously discrete parthenogenetic population of *Tityus serrulatus*, extremely opportunistic, and ‘encouraged’ it to explore the newly created disclimax habitats. The expansion of human colonization towards west and north resulted in a significant regression of the original bisexuals, which were gradually replaced by the parthenogenetic population.

One of the characteristics of opportunistic and parthenogenetic species is their density-independent population regulation, whereas equilibrium species show density-dependent population regulation. This is the reason why, in both old and recent literature describing scorpionism in Brazil, we find the expression of ‘population explosions’, corresponding to the local word *surtos* (from the Latin *surctu* = > *surrectu*: ‘to appear’), which reflects the type of population fluctuation observed in *T. serrulatus*.

The mechanism of selection of species that have low ecological plasticity acts indistinctly on noxious and innocuous species. Ecological plasticity is not associated with the presence of toxic venom. Numerous noxious equilibrium species are selected negatively by human activities on the environment.

The positive selection of noxious opportunistic species is directly associated with human activity. Let us suppose that, in a profoundly modified environment, as in several ‘artificial’ cities of Brazil, the human population begins to increase rapidly. Within a short period, the three main factors needed to transform the region into an important center of scorpionism are assembled:

1. Large-scale demographic expansion of the human population.

2. Rapid expansion of the noxious opportunistic scorpion population that will soon occupy all the empty niches left by the regression or extinction of the equilibrium species. In many cases, the opportunistic species changes its behavior and begins to live inside human dwellings.

3. The overlapping of a large human population with a large population of noxious scorpions will increase enormously the probability of incidents of scorpionism. This situation is characteristic of certain regions in Brazil.

The parthenogenetic *T. serrulatus* usually colonizes urban areas (cities and towns), and can easily be transported by human agency from old to new cities. Brasilia, for instance, has been invaded and was colonized by *T. serrulatus* in less than 15-20 years [67]. What has been observed in the region of Brasilia is certainly also taking place in other cities and towns of different regions in Brazil (Fig. 6).

Curiously, however, the absolute number of noxious species seems to remain stable. Over a period of many years, several authors have suggested that only a limited number of species can be really dangerous to humans [68]. I suggest that this number is, in fact, much higher in nature, but that many noxious species are never in close contact with humans. In very large natural regions such as Amazonia, human populations were scarce for centuries, and only in recent years a rapid expansion of colonization has occurred, with dramatic destruction of the natural environment. It is quite possible that several species of the genus *Tityus* in Amazonia may be highly toxic. Isolated cases of lethal incidents have been observed after stings by *Tityus metuendus* Pocock and *Tityus obscurus* Gervais [69]. Such as many others, however, these species are probably equilibrium species and will be partially or completely selected by the destruction of their environment before human populations become large and vulnerable to them.

**Fig. 7:**
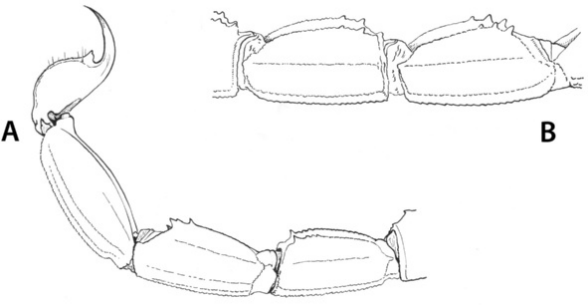
Serrulas present on the dorsal carinae of metasomal segments of *Tityus* species: (**a**) *Tityus kuryi* Lourenço and (**b**) *T. serrulatus*

Morphologically, *Tityus serrulatus* is well known. The following general description is proposed: scorpions of medium size ranging from 55 to 70 mm in total length, general coloration yellowish with the presence of dark confluent spots over the tergites. Metasomal segments I to V are yellowish with lateral and ventral areas blackish. The dorsal keels of segments III and IV have the posterior granules modified as spines, which vary from 4 to 6 in number. These form a serrula (Fig. 7) which is the origin of the Latin name of the species, *T. serrulatus*. Dentate margins of pedipalp-chela fingers are composed of 13 to 17 oblique rows of granules. Telson has a long curved aculeus; the subaculear tooth is strong and spinoid. Pectines have 18 to 25 teeth.

Geographical distribution: the species is found in the southeastern and central states of Brazil: Minas Gerais, São Paulo, Goiás, Federal District and possibly Mato Grosso and Rondônia. It was also recorded in Argentina and Bolivia.

## *Tityus costatus* (Karsch, 1879)

**Fig. 8:**
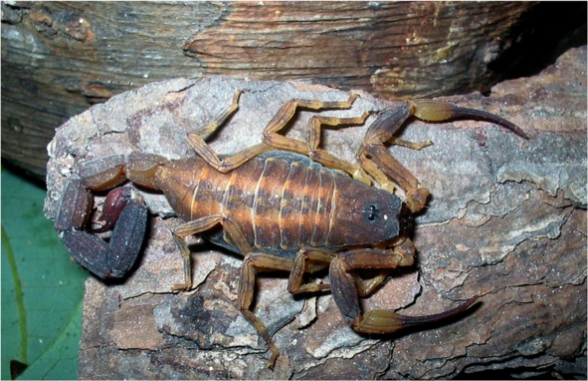
Female of *Tityus costatus, trifasciata* form from the state of São Paulo (photo courtesy of T. J. Porto)

*Tityus costatus* (Fig. 8) was described by Karsch [70], based on specimens collected in the state of Rio de Janeiro. Although this species does not have an infamous reputation, it may be responsible for several incidents in the coastal regions of Brazil. These incidents are certainly misidentified and, in several cases, attributed to *T. bahiensis* or *T. serrulatus*. Not much is known about the venom or toxins produced by this species. Nonetheless, in an already old publication Bücherl and Pucca [71] suggested that it could have a strong effect on mice.

In their study on *Tityus serrulatus* venom, Chavez-Olórtegui et al. [72] refer to an interesting historical description, concerning the first record of scorpion envenoming in Brazil. This was described by Jean de Léry [73], a French Calvinist who took part in the French expedition – commanded by André Villeigagnon – that aimed to establish a colony in Brazil. This colony should be ‘La France Antarctique’, and the group arrived at the Guanabara Bay, today Rio de Janeiro. According to Prof. Carlos R. Diniz (who presented a communication in the 1^st^ International Congress on Envenomations and Their Treatments, which took place in the Pasteur Institute, Paris, in 1995), the scorpion responsible for this incident could only be *Tityus serrulatus*.

The description by Jean de Léry is rather vague and could be associated with different species of scorpions. However, having into account the described symptoms, one can presume that the incident was caused by a buthid scorpion. The description of J. de Léry can be summarized as follows: ‘In Brazil, by moving the stones or grabbing the ground, one can find scorpions which, even if smaller than those found in Provence [he was probably referring to *Buthus occitanus* Amoreux] have their venomous and deadly aculeus, as I could experience myself. The animal seems to be attracted to clean objects. After I had washed my cotton hammock, I prepared to lay down as the natives do, without noticing that a scorpion was hidden in the lining. It stung me on the thumb of my left hand, which swelled rapidly. The venom would have spread throughout my body if I had not been treated by an apothecary, who preserved scorpions in a bottle containing oil. Despite the remedy, reputed to be the most effective in this case, I was in such a state for twenty-four hours that I could not stand the terrible pain. When the local savages are stung by a scorpion, they use the same remedy, which means they kill the scorpion and immediately squash it on the stung area’ [73].

For a better illustration, the original description made by Jean de Léry in Old French is included herein:

J’adjousteray encores, qu’en remuant la terre et dessous les pierres, en nostre contrée du Bresil, on trouve des scorpions lesquels, combien qu’ils soyent beaucoup plus petits que ceux qu’on voit en Provence, neantmoins pour cela ne laissent pas, comme je l’ay experimenté, d’avoir leurs pointures venimeuses et mortelles. Comme ainsi soit doncques que cest animal cerche les choses nettes, advint qu’apres que j’eu un jour fait blanchir mon lict de cotton, l’ayant rependu en l’air, à la façon des sauvages, il y eut un scorpion qui s’estant caché dans le repli: ainsi que je me voulu coucher, et sans que je le visse, me picqua au grand doigt de la main gauche, laquelle fut si soudainement enflée, que si en diligence je n’eusse eu recours à l’un de nos Apothicaires (lequel en tenant de morts dans une phiole, avec de l’huile, m’en appliqua un sur le doigt), il n’y a point de doute que le venin ne se fust incontinent espanché par tout le corps. Et de fait, nonobstant ce remede, lequel neantmoins on estime le plus souverain à ce mal, la contagion fut si grande, que je demeuray l’espace de vingt-quatre heures en telle destresse, que de la vehemence de la douleur je ne me pouvois contenir. Les sauvages aussi estans piquez de ces scorpions, s’ils les peuvent prendre, usent de la mesme recepte, assavoir de les tuer et escacher soudain sur la partie offense [73]*.*

Most obviously, the incident described by J. de Léry cannot be attributed to *T. serrulatus*, which was not a native element of the Rio de Janeiro region on the 16^th^ century. Only several centuries later this species colonized this part of Brazil.

Instead, *Tityus costatus* is an autochtonous element of the Brazilian Atlantic forest, and most certainly was a common species in the area when the French expedition took place, from 1555 to 1558. Consequently, this species is the perfect suspect of being the responsible of the first reported scorpion incident in Brazil.

Morphologically, *Tityus costatus* is a rather poorly known species by non-experts. For a long time it remained inadequately characterized. Lourenço [15] stated that it was difficult to distinguish *T. costatus* from *Tityus trivittatus dorsomaculatus* Lutz & Mello. Studies on additional material from several localities in the Atlantic Forest, from the states of Espírito Santo to Rio Grande do Sul, establish the existence of a mosaic pattern of polymorphism in *Tityus costatus* [32]. The two nominal species are only different morphs of one single species. *T. costatus* is defined as a *maculata* form and *T. dorsomaculatus* as a *trifasciata* form.

The following brief description of its distinguishing characteristics is offered: scorpions of medium size, from 50 to 70 mm in total length. The general coloration is yellowish brown with three longitudinal stripes over the tergites in the *trisfaciata* form or with blackish tergites in the *maculata* form. Metasomal segments I to III are yellowish; IV and V are reddish; and presence of strong spots ventrally. Pedipalps are yellowish to reddish-yellow and strongly spotted; fingers are very dark. Legs are yellowish with diffuse fuscous spots. Dentate margins of pedipalp-chela fingers are composed of 15 to 17 oblique rows of granules. Telson has a long curved aculeus; subaculear tooth is strong and spinoid. Pectines have 16 to 20 teeth.

The geographical distribution includes the states of Espírito Santo, Minas Gerais (‘Zona da Mata’), São Paulo, and Paraná to Rio Grande do Sul. The *maculata* form is generally found in altitudes above 1000 m, whereas the *trifasciata* form occurs at lower altitudes down to the sea level (Fig. 2).
